# Adult lifespan normative data (18–92 years) for executive function tests; the Stroop colour word test, COWAT and Hayling sentence completion test

**DOI:** 10.1111/jnp.70001

**Published:** 2025-06-26

**Authors:** Patrick Murphy, Emily Webster, Lisa Cipolotti

**Affiliations:** ^1^ Department of Neuropsychology National Hospital for Neurology and Neurosurgery London UK

**Keywords:** COWAT, executive functions, Hayling, normative data, older adults, Stroop

## Abstract

The neuropsychological assessment of executive functions is an important part of the diagnostic process for many neurological diseases and for predicting the ability of neurological patients to function independently. Unfortunately, for the majority of commonly used executive function tests there is a paucity of updated normative data, particularly for older adults. This complicates the process of a clinically meaningful assessment. To help address this, we provide normative data for three well‐validated tests of executive functions, the Stroop Colour/Word Test, the Controlled Oral Word Association Test and the Hayling Sentence Completion Test, alongside scores from an estimate of general intellectual ability. These tests are sensitive to frontal lobe damage and provide clinicians with information about possible focal damage to the left and right frontal lobes. Percentiles are presented for five age cohorts across the adult lifespan (18–92 years). A regression equation with age and predicted full‐scale IQ also allows for the categorisation of normal and defective performance on the Stroop and Hayling tests. Given the increasing proportion of older adults requiring neuropsychological assessment, we investigated separately two groups in the older adult range: 65–79 years and 80–92 years. We found a decline in performance for older adults on all three tests. This decline was more marked amongst lower scoring older adults. We did not find a significant relationship between sex and performance on any of the three tests. The findings are discussed in the light of the cognitive reserve theory of ageing.

## INTRODUCTION

### Normative data for executive function tests

Executive functions are higher level cognitive processes that are crucial for adaptive behaviour in humans. Definitions of executive functions usually include the ability to initiate and sustain a response, the ability to monitor ongoing behaviour, the ability to inhibit and switch adaptively between responses, and the ability to plan and strategise (Shallice & Cipolotti, 2018; Stuss & Alexander, 2000). At a diagnostic level, deficits in executive functions are key features of neurological conditions such as Parkinson's disease (Kudlicka et al., 2011), motor neuron disease (Neary et al., 2000) and multiple sclerosis (Foong et al., 1999), as well as being a common sequalae of frontal lobe lesions resulting from stroke, brain tumours, and traumatic brain injury (Stuss & Alexander, 2000). At a functional level, deficits in executive functions predict problems with academic and occupational attainment, problems with mental and physical health, interpersonal difficulties, and difficulties functioning independently (Diamond, 2013; Marshall et al., 2011). Thus, the ability to accurately identify executive function deficits has implications for the diagnosis, treatment, and rehabilitation of neurological conditions.

The usefulness of our assessment of executive functions is limited by the quality of normative data used to classify test performance. A single neuropsychological test score compared with different reference populations can yield classifications ranging from average to impaired (Kalechstein et al., 1998). Also, it is well known that test performance on psychometric tests in the general population has improved over time, in particular for tests tapping frontal lobe functioning (Neisser, 1997). Considering this, our ability to validly interpret test scores will be boosted if the reference sample is the same for each test used and the data have been gathered from contemporary cohorts (see Strauss et al., 2006, pp. 45–46 for discussion). Given the dissociation of different aspects of executive functions based on the location of frontal lobe damage (see Shallice & Cipolotti, 2018 for review), an additional criterion is that the dataset would cover an appropriate range of executive function tests.

Normative data must also respond to changes across the lifespan, which is of particular importance when testing executive functions. The age of onset of neurodegenerative conditions that specifically affect the frontal lobes is quite broad (Moore et al., 2020; Pagano et al., 2016). Thus, data sets that cover a limited age range will be less helpful in tracking disease progression. Numerous empirical studies have indicated that older age is associated with a decline specifically in scores on executive functions (see Salthouse et al., 2003 for extensive review). For example, increasing age is associated with a decline in planning and sequencing abilities (Allamanno et al., 1987), the ability to maintain or shift set (Axelrod & Henry, 1992) and verbal fluency (Souchay et al., 2000). There is also evidence that the age‐related decline in executive functions is more pronounced than the decline in other areas of cognition. Whelihan and Lesher (1985) demonstrated that response inhibition declined with increasing age in a sample of older adults, with core language abilities and praxis relatively well‐preserved. Older age was associated with an increased number of perseverative errors in a further study, with performance on visuo‐spatial tasks preserved (Libon et al., 1994). These findings accord with the “frontal ageing hypothesis”, which predicts that the frontal lobes are especially vulnerable to the effects of normal ageing (see Greenwood, 2000 and West, 2000 for discussion). This hypothesis has further support from studies demonstrating a pronounced age‐related decline in fluid reasoning abilities associated with frontal lobe function (Murphy et al., 2023), versus a relative preservation of crystallised abilities more dependent on the wider cerebral cortex (Murphy et al., 2020).

Examining recently published (since 2000) normative data for executive function tests in English‐speaking populations shows that few datasets address the issues discussed above. Many studies present data for one or a limited range of executive function tests (Bielak et al., 2006; Loonstra et al., 2001; Morrow, 2013; Troyer et al., 2006; Van Der Elst et al., 2006). This requires the use of data sets from alternative reference populations for other administered executive function tests, raising the risk of misclassifying scores. Some studies present data for tests that provide limited sensitivity to focal damage across the entire frontal lobes. For example, Bielak et al. (2006) presented data for a respectably sized sample of older adults who completed the Hayling sentence completion test (HSCT), but no data for tests that cover functions associated with the left frontal lobe (e.g. phonemic fluency, Cipolotti et al., 2020). Furthermore, the second test for which data were presented, the Brixton Spatial Anticipation Test, is relatively insensitive to deficits associated with frontal lobe lesions (Mole et al., 2020). Similarly, Salthouse et al. (2003) presented data for a wider range of executive function and non‐executive function tests including general intellectual ability for 261 adults between the age of 18 and 84. However, of the five executive function tests used, the Wisconsin Card Sorting Test (Anderson et al., 1991), the trail‐making test (Chan et al., 2015) and design fluency (Cipolotti et al., 2020) show poor sensitivity to frontal or posterior cortical lesion location, thus providing limited utility for diagnostic clinical questions. Other studies have provided data with reference samples that cover only younger and middle‐aged adults (De Luca et al., 2003; Hankee et al., 2016; Morrow, 2013), middle‐aged and older adults only (Bielak et al., 2006; Steinberg et al., 2005), or older adults only (Anstey et al., 2000; Zhou et al., 2023). A further issue is the absence of an estimate of general intellectual ability from some studies (Tremblay et al., 2016; Van Der Elst et al., 2006), which would allow clinicians to compare a patient's performance with their premorbid intellectual baseline.

### Current study

The goal of the current study was to provide a normative data set for executive function tests that (i) allows an assessment of a range of executive functions, (ii) provides sensitivity to focal compromise across the entire frontal lobes, and (iii) allows tracking of a decline across the entire adult lifespan. To address this, we present age‐stratified normative data for a group of healthy subjects aged between 18 and 92 years. Data are presented in age cohorts to allow for the calculation of percentiles to evaluate scores (Lezak et al., 2012). Given the known limitations of this approach (Van Breukelen & Vlaeyen, 2005), we also present a method of calculating a defective score based on a multiple regression method, with demographic variables and estimated intellectual level as predictors.

The executive function tests were a test of phonemic fluency (COWAT), which shows sensitivity to left frontal lobe lesions (Cipolotti et al., 2020), The SCWT, a test of inhibition which also shows sensitivity to left frontal lobe lesions (Cipolotti, Spanò, et al., 2016), and the HSCT, which implicates inhibition and strategy formation and shows sensitivity to right frontal lobe lesions (Cipolotti, Healy, et al., 2016; Volle et al., 2012).

## MATERIALS AND METHODS

### Participants

All participants (*N* = 453) were recruited from a neuropsychology department within a hospital in England. The hospital is located within a diverse urban environment with a relatively high proportion of residents from either White ‘other’ ethnic groups (White but not British) or from black, Asian, mixed or other ethnic groups. Relatives and friends of patients who attended the service were approached either directly or via telephone. Employees of the hospital from outside the neuropsychology department were approached directly. Friends and relatives of members of the department were also approached either directly, via telephone or email.

As per our previous studies (e.g. Murphy et al., 2023) and the recommendations of Strauss et al. (2006, pp. 49), we collapsed our sample into age bands with narrower ranges where more marked age‐related changes are expected. Five age groups were created with an interval of 17 years in the youngest cohort (18–34 years, *N* = 97), intervals of 15 years in middle‐aged and “younger” older adults (35–49 years, *N* = 96; 50–64 years, *N* = 128; 65–79 years, *N* = 86) and 10 years in the oldest cohort (80–82 years, *N* = 46). Due to time constraints, not all participants completed every test. The overall numbers completing each test for each age cohort are detailed in Tables 4a, 4b, 5, 6a and 6b below.

The study was approved by the local Joint Research Ethics Committee. All participants read the study information sheet and provided written informed consent.

The exclusion criteria were: (i) history of neurological disorder, (ii) history of severe or enduring psychiatric disorder, (iii) history of learning disability, either self‐reported or as demonstrated by a score below the 5th percentile on the test of premorbid intelligence, (iv) history of acquired or developmental language or perceptual disorder as per self‐report.

Additionally, patients for whom English was a second language and who did not complete their education through English were not asked to complete the HSCT, the National Adult Reading Test (NART), or the phonemic fluency task. All participants who completed the Stroop colour word test had manifestly fluent English.

### Materials

#### Stroop colour word task (SCWT)

In the SCWT (Stroop, 1935), subjects are presented with a list of colour words. Each word is printed in a colour of ink that is incongruent with the printed word (e.g. “Blue” printed in red ink). In part one of the test, subjects are instructed to read the printed words. In part two of the test, subjects are instructed to name the colour of the ink. Subjects are asked to work as quickly and accurately as possible and to self‐correct any errors. Thus, completion time and error rate are measures of performance. Healthy subjects report that part 2 requires inhibition of an automatic response to read the printed word. Thus, an accordingly longer completion time and higher error rate is seen for part 2 (see MacLeod, 1991 for review).

In the current study, the version of the Stroop Test devised by Trenerry et al. (1989) was used. This consists of 112 stimuli for each condition arranged in four columns on one page. The colour words/ink colours Blue, Green, Tan, and Red were used. The number of correct items in 120 seconds and the number of uncorrected errors were recorded for the incongruent/ink‐naming trial. For subjects completing the 112 items in under 120 seconds, their score was prorated to reflect their expected number of items in 120 seconds.

#### Phonemic fluency (COWAT)

We based our procedure for this test on those of Benton (1968). Subjects were asked to produce as many words as possible beginning with the letter “s” in 1 minute. Subjects were directed to not produce names of people or places, to not repeat words and to not produce multiple words from the same word stem (e.g. slip, slips, slipping). The number of words produced were recorded.

#### Hayling sentence completion test (HSCT)

We administered the HSCT as per the author's instructions (Burgess & Shallice, 1997). In Section 1, the subjects orally completed a series of sentences with one word in a sensible fashion. In Section 2, subjects orally complete a series of sentences like those in Section 1, except they were instructed to provide a word completely semantically unconnected to the sentence (e.g. completing “His leaving home amazed all his …” with “banana”). We recorded the scaled scores for Section 2: response time and error score (type A flagrant errors and type B subtle errors combined).

#### National Adult Reading Test (NART)

The NART (Nelson, 1982) was administered as a brief measure of estimated general intellectual ability (Bright et al., 2018). The NART consists of 50 irregularly spelled words that cannot be pronounced by applying regular grapheme to phoneme spelling rules. The test was administered as per the guidelines from the manual (Nelson, 1982, p. 5). The number of errors committed was converted into an estimated full‐scale IQ score (NART IQ) for each participant using Nelson's data (Ibid, p. 12).

### Procedure

Each participant completed the executive function tests after reading the information sheet and completing the consent form. Testing took place either in the neuropsychological department of the hospital or in a quiet space within the participant's home.

### Statistical analysis

#### Data tabulation

The data from the executive function tests, along with gender, age, and years of education, were tabulated and analysed using the R Programming language via the RStudio interface (Version 2023.12.0.369, RStudio Team, 2020).

#### Percentile analysis

Our first analysis facilitates the commonly used method of using Z‐scores and percentiles derived from raw data to identify defective performance (e.g. Wechsler, 2008). We collapsed our sample into age cohorts as described in Section 2.1 above and calculated means, standard deviations and percentile cut‐offs for each age cohort. Data were tested for normal distribution using Kolmogorov–Smirnoff and Shapiro–Wilk tests and by inspection of Q‐Q plots and histograms. Outliers were identified with a Z‐score above or below ±3.

Statistical tests were used to ascertain significant age cohort differences using parametric tests (one‐way ANOVA and *t*‐tests with Bonferroni post‐hoc test) or non‐parametric tests (Kruskal–Wallis, Mann–Whitney U, Dunn post‐hoc comparisons) where appropriate.

Parametric tests (Pearson correlation coefficient) and non‐parametric tests (Spearman's rho) were used where appropriate. The strength of correlations was interpreted using a rule of thumb (Hinkle et al., 2003).

A significance level of *p* = .05 was adopted.

#### Multiple regression analysis

The preceding analysis has a number of disadvantages as described by Van Breukelen and Vlaeyen (2005). Variables besides age may predict a subject's score, and these should be accounted for in the analysis. Furthermore, when using age cohorts: (i) the sample size is reduced and (ii) subjects close to the limit of an age cohort are compared with a sample that may be much younger or older. The following analysis aimed to address these shortcomings.

Our methodology here is based on that of Van Breukelen and Vlaeyen (2005). Two multiple regression analyses were conducted, with SCWT and COWAT scores as the dependent variable in each case. Age, gender, and NART IQ were taken as a priori predictors. Years of education was not used a predictor given its likely strong correlation with NART IQ (Crawford et al., 1988), given that participants were educated in different education systems and given that NART IQ has been previously shown to be a more robust predictor of age‐related cognitive decline (Boyle et al., 2021; MacPherson et al., 2017). A hierarchical regression was used with age, NART IQ, and gender entered in that order.

Potential outliers were identified where the standardised residual was above/below ±3 based on sample size. Such cases were excluded from the analysis if further diagnostic statistics indicated an undue influence on the model (Cooks distance and hat values).

Multicollinearity between predictors was assessed using variance inflation factor (VIF), with scores above 10 interpreted as significant (Myers, 1994). The independence of residuals was tested using the Durbin–Watson test. Normal distribution of residuals was assessed using the Kolmogorov–Smirnoff and Shapiro–Wilk tests and by examining Q–Q plots and histograms. Linear and quadratic trends for age and NART IQ were compared using an ANOVA. Homoscedasticity was assessed using White's test.

Based on the final regression model, the predicted score Y on an executive function test for any subject was calculated as follows:

(1) $$Y=b_{0}+b_{1}X_{1}+\ldots +b_{n}X_{n}$$

where *X*_1…*n*_ are the significant predictors, *b*_0_ is the intercept and *b*_1…*n*_ are the regression coefficients. Based on this, the patient's standardised residual *Z* can be calculated as follows:

(2) $$Z=\frac{\text{Observed test score}-\text{predicted test score}\;\left(Y\right)}{\mathrm{SD}\left(r\right)}$$

where SD(r) is the standard deviation of the residuals. Based on widely used rules of thumb (Wechsler, 2019), resulting Z‐scores can be categorised based on cut‐offs at the 25th, 10th and 5th percentiles. Thus, a Z‐score >−.67 was categorised as *normal*, a Z‐score between −.67 and −1.28 was *weak*, a Z‐score between −1.28 and −1.65 was *likely defective*, and a Z‐score less than −1.65 was *defective*.

#### Correlation analysis

Given the theoretical interest in the relationship between cognitive reserve proxies such as educational level and performance on executive function tests (MacPherson et al., 2020), we calculated the correlation between age of education and performance on the SCWT, COWAT, and HSCT. Similarly, given the likely clinical interest in the concurrent and divergent validity of the executive function tests (Strauss et al., 2006, p. 18), correlations between the SCWT, COWAT, and HSCT were also calculated.

#### Sex differences

Given the clinical and theoretical interest in sex differences on executive function tests (e.g. Grissom & Reyes, 2019), the performances of males and females on the SCWT, COWAT, and HSCT were compared.

## RESULTS

### Demographic data

Of the 453 participants, 264 were female. Group means from the NART indicated that this was a high‐functioning sample, with mean NART IQ score well above the population mean and in the high average range for all but the 18–34 years cohort (see Table 1).

**TABLE 1:** Demographic data for sample.

Cohort		Age/years	NART IQ	Years of education
18–34 years	Mean	26.55	106.76	15.67
	SD	3.97	9.62	1.98
35–49 years	Mean	42.22	110.13	15.04
	SD	4.54	10.11	2.77
50–64 years	Mean	57.1	112.99	14.8
	SD	4.3	10.7	2.93
65–79 years	Mean	70.64	114.07	13.46
	SD	4.59	9.78	2.78
80–92 years	Mean	83.72	113.17	13.25
	SD	3.69	11.96	3.38

Abbreviation: NART, National Adult Reading Test.

A statistical test showed that the age cohorts differed in terms of NART IQ, *F*(4,414) = 7.352, *p* < .001. Inspection of the means for each age cohort (See Table 1) showed a trend towards a higher NART IQ in older age cohorts. The age cohorts also differed in terms of age of education *F*(4, 369) = 9.59, *p* < .001. The means for each age cohort (see Table 1) indicated a trend towards more years of education in younger age cohorts.

### Correlations between executive function tests and years of education

Table 2 depicts the correlations between the three executive function tests. All correlations were low, aside from COWAT and HSCT Section 2 time, where no or little correlation was noted. Years of education showed a low correlation with SCWT (*r* = .352), COWAT (*r* = .411) and HSCT Section 2 errors (ρ = .309). Years of education and HSCT Section 2 time did not correlate (ρ = .183). Age showed a low correlation with HSCT Section 2 time (*r* = .369) and error score (*r* = .484). NART and years of education did not correlate with the HSCT.

**TABLE 2:** Correlations between neuropsychological tests.

	COWAT(S)	SCWT	HSCT Section 2 time
SCWT	.490^a^		
HSCT Section 2 Time	.204	.386^a^	
HSCT Section 2 Errors	.307^a^	.418^a^	.382^a^

Abbreviations: COWAT, Controlled Oral Word Association Test; HSCT, Hayling sentence completion test; SCWT, Stroop colour word test.

^a^ Low correlation.

### Percentile analysis: Results

#### Screening data

One participant with a Z‐score of +3.97 for the SCWT was excluded from the analysis. Inspection of Q–Q plots, histograms and Kolmogorov–Smirnov/Shapiro–Wilk test results for the HSCT showed a significant divergence from normality for Section 2 Response Time (*W* = .765, *p* < .001), Section 2 Category A errors (*W* = .614, *p* < .001) and Section B errors (*W* = .832, *p* < .001). The Shapiro–Wilk test was significant for the COWAT (*W* = .989, *p* = .020) and the SCWT (*W* = .987, *p* = .017). However, inspection of the histograms and Q–Q plots indicated minimal deviation from normality. Thus, the data for these two tests were treated as normally distributed, with the significant results likely a by‐product of the large sample size (Mishra et al., 2019).

#### Normative data

Table 3 details the descriptive statistics for the age cohorts on the executive function tests. Tables 4a, 5, and 6a detail the percentile cut‐offs for each cohort on the tests. Given the non‐parametric and skewed nature of the data from the HSCT, cumulative percentages are presented rather than percentiles. Table 3 can be used to calculate Z‐scores based on a subject's test score or alternatively the test score can be compared with the cut‐offs in Tables 4a, 4b, 5, 6a and 6b. This allows an assessment of whether a subject's score is so uncommonly low that it is likely abnormal. For example, a score below the 5th (*Z* = −1.64) or 2nd (−2.05) percentiles is commonly denoted as an *impaired* score (Wechsler, 2008).

**TABLE 3:** Means and standard deviations from executive function tests.

Cohort		SCWT^a^	HSCT Section 2: time^b^	HSCT Section 2: errors^b^	COWAT: S^c^
18–34 years	Mean	130.69	6.24	7.16	17.72
	SD	24.21	0.59	1.28	4.51
35–49 years	Mean	119.25	6.04	6.96	17.1
	SD	20.71	0.74	1.22	4.78
50–64 years	Mean	108.13	5.88	6.42	18.06
	SD	25.81	0.6	1.58	5.16
65–79 years	Mean	96.77	5.71	5.75	16.03
	SD	30.25	0.93	2.1	5.05
80–92 years	Mean	70.13	5.51	4.74	14.14
	SD	30.26	1.18	2.19	5.75

Abbreviations: COWAT, Controlled Oral Word Association Test; SCT, Hayling sentence completion test; SCWT, Stroop colour word test.

^a^ Prorated number of correct responses in 120 seconds shown.

^b^ Scaled scores shown.

^c^ Number of correct responses in one minute shown.

**TABLE 4A:** Percentiles for the SCWT by age cohort: Number of items correct in 2 min.

Percentile	18–34 years	35–49 years	50–64 years	65–79 years	80–92 years
	*N* = 58	*N* = 65	*N* = 68	*N* = 49	*N* = 38
5th	99	92	80	56	25
10th	107	95	85	68	28
25th	113	107	93	84	49
50th	131	118	106	92	69
75th	148	132	123	110	91
90th	162	146	141	128	112
95th	173	150	151	145	117

Abbreviation: SCWT, Stroop colour word test.

**TABLE 4B:** Frequencies (cumulative percentages^a^) of subjects making specified numbers of errors^a^ on the SCWT.

Number of errors	18–64 years	35–49 years	50–64 years	65–79 years	80–92 years
0	86.21	76.56	79.41	69.05	66.67
1	94.83	90.63	86.76	90.48	79.49
2	96.55	92.19	98.53	–	82.05
3	–	100	100	92.86	–
4	98.28	–	–	100.00	84.61
5	100.00	–	–	–	89.74
6+	–	–	–	–	100.00
10% cut‐off^b^	1 (error)	1	–	1	–
5% cut‐off^b^	2 (errors)	3	2	4	6

Abbreviation: SCWT, Stroop colour word test.

^a^ The percentage of subjects making the specified number of errors or less on the SCWT.

^b^ For each cut‐off score, the subject is within this % of subjects at the bottom of their cohort group.

As expected, there appeared to be a trend towards a poorer performance in older age cohorts on the executive function tests. Inspection of Table 3 indicates that the decline for the older adult cohorts is less marked on the COWAT and there is also a generally greater variability in this age range across all tests.

In line with our previous studies (e.g. Murphy et al., 2023), we statistically tested the hypothesis of an age‐related decline in scoring on the executive function tests amongst the two older adult cohorts. To this end, we compared both older adult cohorts (65–79 years and 80–92 years) with each other and we also compared each of these older adult cohorts with the younger cohorts combined as one group (i.e. 18–64 years). These three groups differed significantly in terms of SCWT performance *F*(2, 264) = 55.21, *p* < .001. Using Bonferroni post‐hoc tests, we found that each older adult cohort performed worse than the 18–64 years group (*p* < .001). Additionally, the 80–92 years cohort performed significantly worse than the 65–79 years cohort. For the COWAT, group differences were again significant, *F*(2,300) = 7.198, *p* < .001. Again using Bonferroni post‐hoc tests, the 80–92 year cohort performed significantly worse than the 18–64 years group (*p* < .001). No other group comparison was significant (*p* > .05).

For the HSCT, non‐parametric tests were used to test for group differences. A significant group difference was found for Section 2 time χ^2^(2) = 17.56, *p* < .001. Post‐hoc Dunn tests showed that the 80–92 years cohort were significantly slower than the 18–64 years cohort, *Z* = 2.923, *p* < .001. The 65–79 years cohort were also slower than the 18–64 years cohort, *Z* = 2.458, *p* = .007. No significant difference was found between the two older adult cohorts *Z* = 1.1603, *p* = .123. For Section 2 errors, significant group differences were also observed χ^2^(2) = 51.170, *p* < .001. Here, the 18–64 years cohort performed significantly better than the 80–92 years cohort (*Z* = 6.880, *p* < .001) and the 65–79 years cohort (*Z* = 2.706, *p* < .001). The 65–79 years cohort also performed significantly better than the 80–92 years cohort on this measure (*Z* = 2.706, *p* = .003).

#### SCWT errors

Table 4b shows the cumulative number of subjects making a specified number of uncorrected errors on the SCWT for each age cohort, along with 5% cut‐off scores. There are two points of note. First, making an error is not uncommon for healthy subjects, for example, one in four subjects from the 35–49 years cohort made at least one error. Second, although the relative lack of errors meant a statistical analysis was not feasible, there again appears to be a decline in performance in the two older adult cohorts. For example, almost 20% of the 80–92 years cohort made more than two errors and almost 10% of the 65–79 years cohort made more than three errors.

#### Cohort sizes

The usefulness of the percentiles derived from the age cohorts is clearly dependent on sample size. In their analysis, Crawford and Howell (1998) demonstrate the issues with this approach when the sample size is less than 50. Some of the age cohorts in our study fall just below this level (two for the SCWT, one for the COWAT and two for the HSCT, see Tables 4a, 4b, 5, 6a, 6b).

**TABLE 5:** Percentiles for the COWAT(S) by age cohort: Words produced in 1 min.

Percentile	18–34 years	35–49 years	50–64 years	65–79 years	80–92 years
	*N* = 59	*N* = 61	*N* = 89	*N* = 62	*N* = 44
5th	12	10	9	9	6
10th	14	11	11	11	8
25th	15	14	15	13	10
50th	18	17	18	15	14
75th	20	20	22	19	18
90th	23	23	24	23	22
95th	25	25	26	23	24

Abbreviation: COWAT, Controlled Oral Word Association Test.

**TABLE 6A:** Percentage cut‐offs for the HSCT by age cohort, Section 2 Total Time: Scaled score.

Percentage cut‐off^a^	18–34 years	35–49 years	50–64 years	65–79 years	80–92 years
	*N* = 62	*N* = 45	*N* = 65	*N* = 56	*N* = 43
5%	5	4	4	–	3
10%	–	5	–	–	–
25%	–	–	5	4	4
50%	–	–	–	5	5
75%	6	–	–	–	–
90%	7	6	6	6	–
95%	–	7	7	7	6

Abbreviation: HSCT, Hayling sentence completion test.

^a^ At each cut‐off score, the subject is within this % of subjects at the bottom of their cohort group.

**TABLE 6B:** Percentage cut‐offs for the HSCT by age cohort, Section 2 Errors (A + B): Scaled score.

Percentage cut‐off^a^	18–34 years	35–49 years	50–64 years	65–79 years	80–92 years
	*N* = 62	*N* = 45	*N* = 65	*N* = 56	*N* = 43
5%	2	3	2	–	–
10%	5	4	4	1	1
25%	6	–	–	4	2
50%	–	6	6	5	4
75%	–	–	–	6	6
90%	–	–	7	7	–
95th	–	–	–	–	7

Abbreviation: HSCT, Hayling sentence completion test.

^a^ At each cut‐off score, the subject is within this % of subjects at the bottom of their cohort group.

To more confidently conclude that a score below a given percentile is indeed abnormal, clinicians may wish to use the following equation for age cohorts with a low N:

(3) $$t=\frac{\text{Score}-\text{population mean}}{\text{Standard deviation}\times \sqrt{\frac{N+1}{N}}}$$

This is essentially a modified *t*‐test, the output of which can be compared with standard statistical tables (e.g. Lindley & Scott, 1996) to assess the significance of the result, with *p* = .05 and *p* = .01 denoting a score in the lowest 5% and 1% of a given cohort.

### Regression analysis

#### Data screening and diagnostics

For the COWAT, the Kolmogorov–Smirnoff test for normality of standardised residuals was non‐significant *D* = .058 (*p* = .276), but the results of the Shapiro–Wilk test suggested a non‐normal distribution *W* = .988, *p* = .0139. However, given that the Kolmogorov–Smirnoff test is thought more appropriate for our sample size (Mishra et al., 2019), and that the data aligned well with the theoretical distribution on the Q–Q plot, the standardised residuals were treated as normal. No outliers with an undue influence on the model was identified. The assumptions of multicollinearity and independence of residuals were met. The results of White's test indicated the assumption of homoscedasticity was met (*W* = 3.59, *p* = .464).

For the SCWT, the Kolmogorov–Smirnoff test was non‐significant but the results of the Shapiro–Wilk suggested a non‐normal distribution (*D* = .053, *p* = .463; *W* = .989, *p* = .046). However, as the data again aligned well with the theoretical distribution on the Q–Q plot, the standardised residuals were treated as normal as described for the COWAT. No outliers with an undue influence were identified and the assumptions of multicollinearity and independence of residuals were met. The results of White's test indicated that the assumptions of homoscedasticity were met (*W* = 2.18, *p* = .704).

For the HSCT, the results of the Shapiro–Wilk test indicated that the distribution of standardised residuals deviated from normality both for errors (*W* = .900, *p* < .001) and time (*W* = .833, *p* < .001), with the Q–Q plots indicating a significant positive skew in the distribution. Thus, the HSCT was not included in the regression analysis.

#### Linear and quadratic trends

**FIGURE 1:**
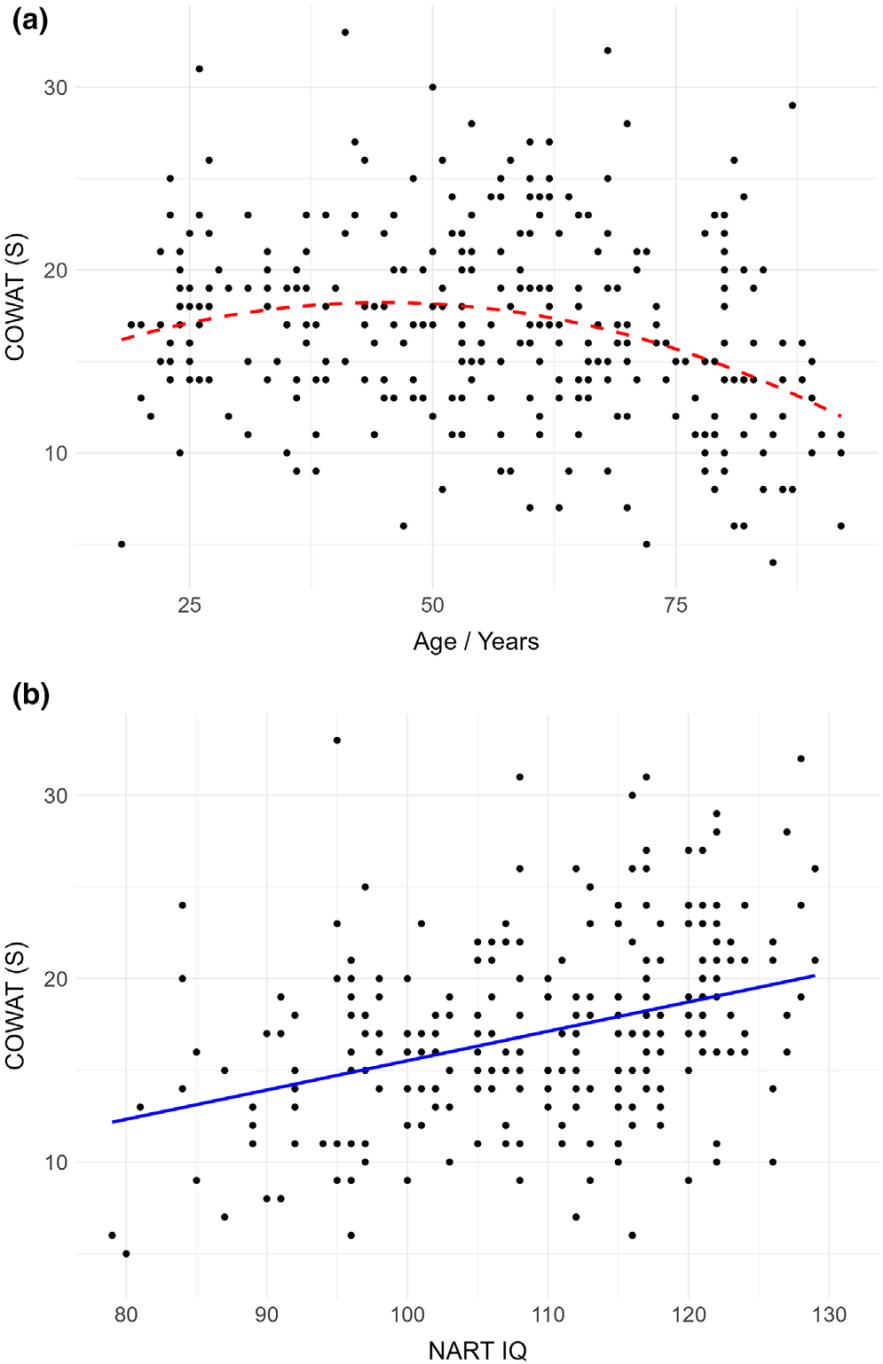
(a) Scatterplot depicting the quadratic relationship between COWAT performance and age. (b) Scatterplot depicting the linear relationship between COWAT performance and NART IQ.

We further explored the relationship between NART, age, and executive function test score by fitting linear and quadratic models to the data and comparing the models using an ANOVA. For the relationship between SCWT and age, a quadratic model showed a better fit than a linear model *F*(2, 276) = 10.898, *p* = .001. This is depicted in Figure 1a; SCWT performance showed a rather gradual decline until middle age, before the decline accelerated. For the relationship between SCWT and NART IQ, the linear model was superior to the quadratic model *F*(2, 256) = 2.317, *p* = .129, with a modest improvement in SCWT score noted with increasing NART IQ (see Figure 1b).

**FIGURE 2:**
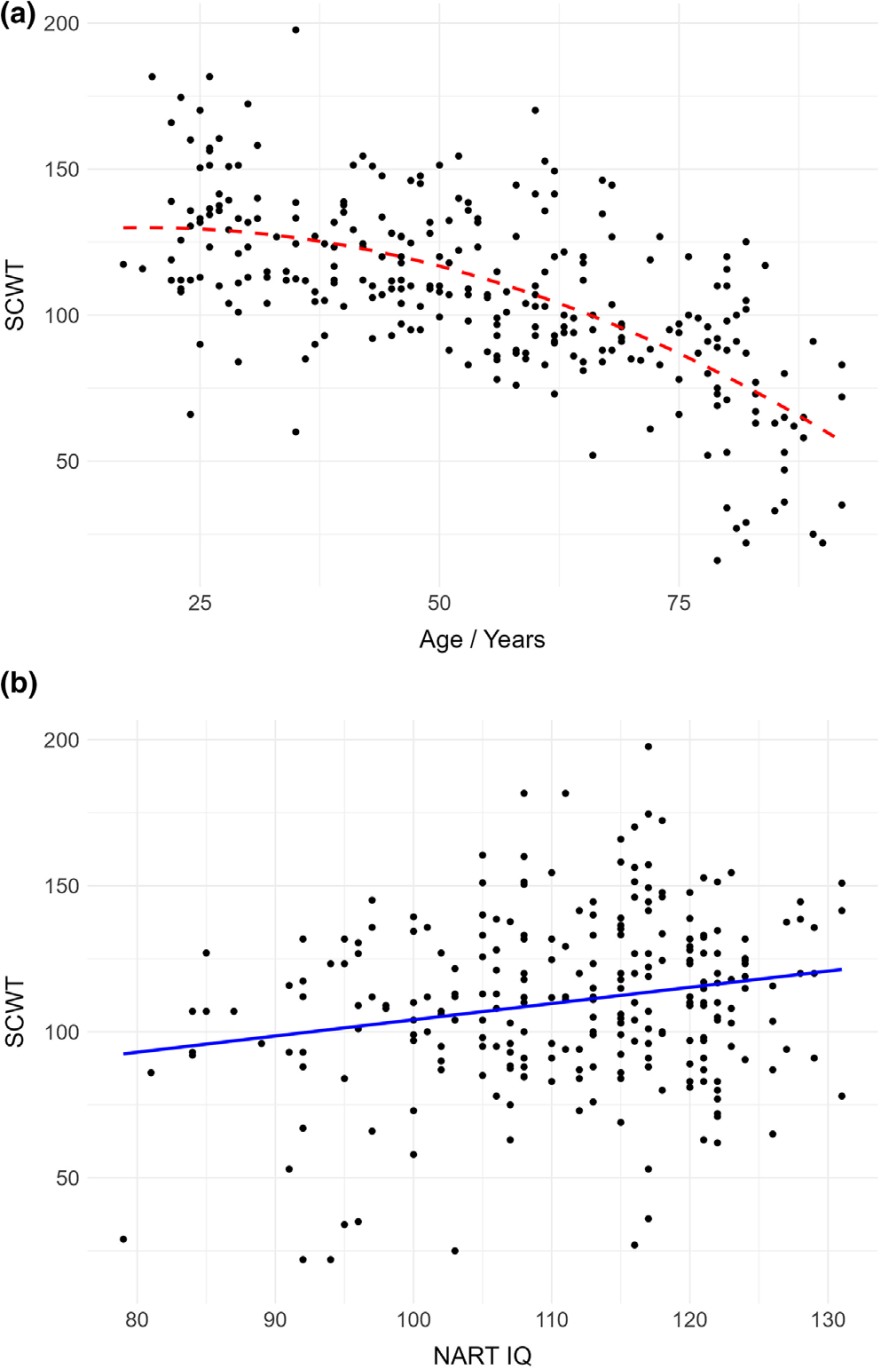
(a) Scatterplot depicting the quadratic relationship between SCWT performance and age. (b) Scatterplot depicting the linear relationship between SCWT performance and NART IQ.

Considering the relationship between COWAT score and age, a quadratic model also showed a better fit than a linear model *F*(2, 311) = 14.273, *p* < .001. Examining Figure 2a, an improvement in performance was evident throughout early adulthood until approximately age 50, with a subsequent decline. In terms of the relationship between COWAT and NART score, the linear model was superior to the quadratic model *F*(2, 288) = .783, *p* = .3769.

#### Predicting SCWT score using a multiple regression

The final model is shown in Table 7. Gender was not predictive of SCWT score (*p* = .554) and was thus omitted from the model. Based on the analysis in Section 3.1.2, a quadratic rather than a linear term for age was a priori included. The final model accounted for a little over half of the variance in SCWT score (*R* ^2^ = .517). The adjusted *R* ^2^ value was very similar (=.513), suggesting good generalisability. Using an ANOVA, we compared the *R*‐squared values of the regression models using linear and quadratic terms. Including the quadratic term significantly improved the variance explained by the final model, *F*(2, 253) = 6.921, *p* = .009. Goodness‐of‐fit indicators are presented in Table 9.

**TABLE 7:** Final regression model for SCWT.

Predictor	B	*SE* B	*p*
Constant	48.95	13.251	.003
Age (Years) squared	−.0094	.0006	<.001
NART IQ	.8190	.1195	<.001

Abbreviation: SCWT, Stroop colour word test.

Using the data in Table 7, we obtained the following equation for calculating the predicted score:

(4) $$\text{Predicted SCWT score}=48.95+\left({\mathrm{Age}}^{2}\times -0.0094\right)+\left(\text{NART}\;\mathrm{IQ}\times 0.819\right)$$

Calculating the standard deviation of the unstandardised residuals, we obtained the following equation for calculating a Z‐score as per Section 2.8.3 above:

(5) $$Z=\frac{\text{Observed SCWT score}-\text{Predicted SCWT Score}}{20.266}$$

To illustrate the utility of this method, we calculated the Z‐score for a 64‐year‐old subject with a NART IQ of 100 who scored 80 on the SCWT. This subject is close to the top of the age range of the 50–64 years age cohort in Tables 3 and 4a. The resulting Z‐score of −.609 is within normal limits. However, comparison of this SCWT score with the percentiles in Table 4a would yield a borderline impaired score.

We also noted that Equations 4 and 5 tend to rate a subject's performance more favourably than the data in Tables 3 and 4a. For example, a subject aged 57 with a NART of 100 and observed SCWT score of 106 is scoring at the 50th percentile as per Table 4a. However, Equations 4 and 5 yield a Z‐score of .281. We interpret this in the light of the high NART IQ sample used to calculate the data in Table 4a, and this is discussed further below.

#### Predicting COWAT score using a multiple regression

The final model is shown in Table 8. Gender was again not predictive of score (*p* = .079) and was omitted from the model, and a quadratic rather than a linear term for age was included. The final model accounted for ~20% of the variance in COWAT score (*R* ^2^ = .191). The adjusted *R* ^2^‐value (=.186) indicated that the model would account for roughly 2.6% less variance within the wider population. Using an ANOVA, we compared the *R*‐squared values of the regression models for the COWAT using linear and quadratic terms. Including the quadratic term significantly improved the variance explained by the final model, *F*(2,287) = 5.245, *p* = .0227. Goodness‐of‐fit indicators are presented in Table 9.

**TABLE 8:** Final regression model for COWAT.

Predictor	B	*SE* B	*p*
Constant	−.890	2.702	.742
Age (Years) squared	−.00064	.0001	<.001
NART IQ	.1833	.0249	<.001

Abbreviation: SCWT, Stroop colour word test.

**TABLE 9:** Goodness‐of‐fit indicators for regression models containing linear and quadratic terms.

		BIC	AIC	MSE
SCWT	Linear	1720.995	1706.316	20.458
	Quadratic	1722.368	1704.019	20.157
COWAT	Linear	2286.088	2271.907	405.658
	Quadratic	2284.404	2266.678	394.363

Abbreviations: AIC, Akaike information criterion; BIC, Bayesian information criterion; COWAT, Controlled Oral Word Association Test; MSE, Mean squared error; SCWT, Stroop colour word test.

Using the data in Table 8, we obtained the following equation for calculating the predicted score:

(6) $$\text{Predicted COWAT score}=-0.89+\left({\mathrm{Age}}^{2}\times -0.00064\right)+\left(\text{NART}\;\mathrm{IQ}\times 0.1833\right)$$

Calculating the standard deviation of the unstandardised residuals, we get the following equation for calculating a Z‐score as per Section 2.8.3 above:

(7) $$Z=\frac{\text{Observed COWAT score}-\text{Predicted COWAT Score}}{4.59}$$

Similar to Section 3.1.2, to illustrate the benefits of this approach, we calculated the Z‐score for a 79‐year‐old subject with a NART IQ of 100 who scored in the impaired range on the COWAT (score = 8) as predicted by the percentiles in Table 4b. This subject is close to the top of the age range of the 65–79 years age cohort. By contrast, the resulting Z‐score calculated using Equations 6 and 7 is −1.186 is categorised as “weak”, thus not entirely convincing of impairment.

Similar to the SCWT, we again noted that Equations 6 and 7 rate subjects' COWAT performance more favourably than the data in Tables 3 and 5. For example, a subject aged 57 with a NART of 100 and observed COWAT score of 18 is scoring at the 50th percentile as per Table 5. However, applying Equations 6 and 6 yield a Z‐score of .575.

#### Regression sample size

We assessed the suitability of our regression sample size using two methods. Using data presented by Miles and Shevlin (2000, pp. 122–125) and given a *p* level of .05 and two predictors, our sample size would be expected to yield a large effect size (>.8). We also examined whether our sample size allowed us to make predictions to the wider population. The squared multiple correlation coefficients were calculated and compared with the data presented from a Monte Carlo simulation by Knofczynski and Mundfrom (2008). The results showed an “excellent” prediction level for the SCWT model (ρ^2^ = .502) and a “good” prediction level for the COWAT model (ρ^2^ = .180).

### Sex differences

Males and females did not differ significantly in terms of years of education *t*(301) = 1.368, *p* = .172 (Males: *M* = 14.844, SD = 3.083; Females: *M* = 14.424, SD = 2.740) or NART IQ *t*(406) = 1.923, *p* = .055 (Males: *M* = 112.33, SD = 9.60; Females, *M* = 110.36, SD = 11.18). Males and females performed similarly on the SCWT, *t*(245) = .270, *p* = .787, and the HSCT Section 2 Time, *W* = 8840.5, *p* = .934, and HSCT Section 2 Errors *W* = 9351.5, *p* = .443. For the COWAT, Males (*M* = 17.535, SD = 5.567) scored slightly higher than Females (Mean = 16.413, SD = 4.871), but this result was narrowly non‐significant *t*(239) = 1.7171, *p* = .067.

## DISCUSSION

We have presented normative data for three executive function tests: the COWAT, the SCWT and the HSCT. As previously discussed, these tests combined provide sensitivity to focal neurological dysfunction across the frontal lobes. The data cover the adult lifespan, including for two cohorts of older adults (65–79 years and 80–92 years). The sample sizes for each age cohort are respectable and generally larger than those from recent similar studies (e.g. De Luca et al., 2003; Libon et al., 1994; Salthouse et al., 2003). Although one previous study presented data from larger samples for each age cohort, that data covered a much narrower range of the adult lifespan (Bielak et al., 2006). Additionally, we have provided data on estimated premorbid intellectual level against which to index any potential decline in patients' scores on the executive function tests. Thus, the current dataset provides clinicians and researchers with a reliable method both of identifying a likely decline in executive functions and tracking any decline across the adult lifespan.

A significant decline in performance was noted for older adults on each of the three tests. Our data are therefore in line with previous studies demonstrating an age‐related decline on tests implicating the frontal lobes, such as fluid intelligence (e.g. Murphy et al., 2023). Some previous studies have attributed a decline on timed executive function tests in older adults to cognitive slowing, a hallmark of cognitive ageing associated with compromised white matter integrity (Kerchner et al., 2012). For example, in their meta‐analysis, Verhaeghen and De Meersman (1998) found that any decline on the SCWT in older adults could be attributed to reading speed. Similarly, a further meta‐analysis by Rey‐Mermet and Gade (2018) found that older adults showed no age‐related decline on most standard tests of inhibition, including the SCWT. In their conclusions, both studies queried the hypothesis of a decline in inhibitory skills in older age. However, neither of these studies presented data on error rates on the SCWT for older adults. Our data demonstrates increased errors on the SCWT and the HSCT that cannot be easily attributed to general ageing effects such as cognitive slowing, thus supporting the hypothesis of a decline in inhibitory skills in older age. Given that such errors are associated with focal frontal lobe lesions (Cipolotti, Spanò, et al., 2016; Stuss et al., 2001), our data also provides support to the hypothesis that frontal lobes are particularly susceptible to the effects of normal ageing (Raz, 2000; West, 2000).

Our analyses provided a more finer grained understanding of the relationship between age and test score. In particular, phonemic fluency (i.e. COWAT score) appeared more preserved amongst older adults. In contrast to the SCWT and HSCT, we found that only the oldest cohort (80–92 years) performed significantly worse than the 18–64 years subjects on the COWAT, with the 65–79 years cohort performing at a similar level to the 19–64 years subjects. Furthermore, COWAT score improved in our sample until middle age before declining. SCWT score by contrast declined from early adulthood, with an acceleration in the decline evident from around age 50. This may be explained by the cognitive factors underpinning performance on the COWAT. As a measure of executive function, phonemic fluency has been demonstrated to also load highly on crystallised abilities such as expressive vocabulary and object naming (Whiteside et al., 2016), which are known to improve throughout early adulthood (Wechsler, 2008) and remain relatively better preserved in older age (Tucker‐Drob et al., 2022).

The age‐related decline on each test was most marked amongst lower scoring subjects. For example (see Tables 4a and 4b), comparing the youngest (18–34 years) and oldest (80–92 years) cohorts on the SCWT, there was a decline of 74% in terms of raw score for those scoring at the 10th percentile, but a decline of only 31% for those scoring at the 90th percentile. An influential hypothesis that may potentially explain this result is an increased cognitive reserve for the older subjects with more years of education, higher intellectual ability and with more lifetime exposure to cognitively enriching experiences (Petrosini et al., 2009). There was some tentative evidence in support of this hypothesis in our data. Compared with the sample as a whole, general intellectual ability and years of education were relatively stronger predictors of better performance on the executive function tests for the older adult cohorts. Regardless of the explanation, our data indicate that even healthy older adults may encounter difficulties with activities of daily living that require good executive functioning. It also emphasises the need for an appropriate comparison group when assessing older adults of relatively lower ability for executive dysfunction.

We have presented two methods of assessing the performance of a subject on the SCWT and the COWAT. The percentile analysis allows for the calculation of Z‐scores and percentiles with respect to a subject's age cohort, a very commonly used strategy in clinical settings in the anglophone world (Lezak et al., 2012; Strauss et al., 2006; Wechsler, 2012). This approach allows rapid assessment of scores with percentiles, which is advantageous in busy clinical settings and for bedside testing. Using percentiles alongside qualitative descriptors also facilitates communication of results and clinical reasoning with non‐specialist medical and therapeutic staff. However, as described above, this method has drawbacks (Van Breukelen & Vlaeyen, 2005). In particular, we have highlighted the issue in using percentiles to assess performance of subjects close to the limit of an age cohort and using this method when comparison data come from a high‐functioning sample. A compromise between these approaches, particularly for those accustomed to the traditional percentile approach, is to also calculate results for subjects close to the limit of an age cohort using the regression equations. This is particularly recommended where results are less obviously below a subject's expected level and where other clinical and physiological signs of neurological compromise are equivocal.

We found no statistically significant difference between males and females on the three executive function tests. These results contradict those from some previous studies. For example, in a narrative review, MacLeod (1991) found no convincing evidence for sex differences in terms of the Stroop effect, but in a more recent study of over 1700 subjects Van Der Elst et al. (2006) found a clear advantage for females in terms of completion time for the SCWT. Similarly, a large meta‐analysis aggregating data from over 300,000 subjects showed an advantage for females in terms of performance on the COWAT. There are two potential explanations for our failure to replicate these results. Firstly, our sample may not have been large enough to reveal the differences noted in these studies. Secondly, although non‐significant, there was a trend towards more years of education and higher NART IQ in the male sample. This confound may have negated any advantage that females have on the COWAT and the SCWT.

Different aspects of executive functions show sensitivity to distinct lesion locations. For example, Cipolotti, Healy, et al. (2016) tested patients with focal frontal lobe lesions on the HSCT. Despite the clear linguistic nature of the task, the critical lesion site was found within the right frontal lobe. Cipolotti, Spanò, et al. (2016) further demonstrated a dissociation between deficient performance on the HSCT and the SCWT, with the critical lesion site for the latter test in the left frontal lobe. Left frontal cortical areas are also well known to underpin performance on the COWAT (Biesbroek et al., 2021; Stuss et al., 1998). In terms of the diagnosis of neurodegenerative conditions, studies have shown that specific executive function tests are sensitive to the executive function deficits in progressive supranuclear palsy (COWAT: Burrell et al., 2014), Alzheimer's disease (SCWT: Spieler et al., 1996) and frontotemporal dementia (HSCT: Hornberger et al., 2008). This body of evidence points to the need for a broad assessment of executive functions in clinical settings. We believe that the dataset presented here will assist greatly with this.

One limitation of our study relates to language. All three tests were linguistic in nature to varying degrees, and are thus less useful for patients with limited or no English. As already discussed, the COWAT in particular correlates strongly with expressive vocabulary. We would thus recommend using this test only with patients who are at least strongly fluent speakers of English, if not native speakers. More generally, our dataset does not include any purely non‐verbal tests. This makes it less useful for the assessment of non‐linguistic executive function deficits in patients with expressive or receptive language problems. Existing non‐verbal executive function tests have conceptual issues, particularly in terms of the level to which they also tap language processes (see Shallice & Cipolotti, 2018, for discussion). Some commonly used non‐verbal executive function tests also show limited sensitivity to frontal lobe damage, including the Brixton Spatial Anticipation Test (Mole et al., 2020), the design fluency test (Marin et al., 2017) and the Trail‐Making Test Part B (Chan et al., 2015). Future studies should provide datasets for non‐verbal tests that provide sensitivity and specificity to frontal executive dysfunction.

A further notable limitation is that although we recruited subjects from a culturally diverse location, we did not collect information on ethnic background. We therefore cannot state with certainty how culturally fair the tests and our dataset are. Therefore, some further caution should be exercised when interpreting any deficits in individuals of non‐Western cultural backgrounds. A further limitation is the lack of an analysis of the test–retest reliability. This reliability data has been presented in previous studies for the SCWT (subjects retested on different test versions: Siegrist, 1997), the COWAT (subjects retested on same test version: Ross et al., 2007) and the HSCT (subjects retested on the same version: Burgess & Shallice, 1997). We also did not examine the validity of the tests in differentiating lesion sites within the brain, although data supporting this has also been previously presented (Cipolotti, Healy, et al., 2016; Robinson et al., 2012).

Our data analysis showed severe skew in HSCT data, which precluded further analysis using demographic predictors and NART IQ. To create smoother and less skewed data, future studies could collect finer grained response times or revisit the weighting for flagrant and subtle error types (Burgess & Shallice, 1997). Also, we found that a relatively low proportion of variance in COWAT score was explained by age and predicted full‐scale IQ. Future studies should thus examine what wider demographic factors or executive function tests predict COWAT score, which could facilitate a more robust regression model. Further modelling of executive function data could also expand on the statistical techniques used here, such as including potential interactions between demographic variables in regression models (Bläsi et al., 2009), or more novel modelling techniques such as the use of general linear models (Rivera et al., 2023).

In summary, the dataset of normative data presented here allows for the identification of executive function deficits across the adult lifespan from ages 18 to 92 years and allows such deficits to be tracked. The data confirms the known decline in executive functions in healthy older adults and the relatively more marked decline in this domain amongst lower scoring individuals.

## AUTHOR CONTRIBUTIONS

**Patrick Murphy:** Conceptualization; methodology; validation; supervision; formal analysis; project administration; writing – original draft. **Emily Webster:** Conceptualization; data curation; resources; supervision; project administration; methodology; writing – original draft. **Lisa Cipolotti:** Conceptualization; investigation; writing – original draft; funding acquisition; methodology; project administration; supervision; resources; validation; writing – review and editing.

## CONFLICT OF INTEREST STATEMENT

The authors declare no conflicts of interest.

## Data Availability

The data that support the findings of this study are available from the corresponding author (LC) upon reasonable request.
